# Niacin therapy and the risk of new-onset diabetes: a meta-analysis of randomised controlled trials

**DOI:** 10.1136/heartjnl-2015-308055

**Published:** 2015-09-14

**Authors:** Christina Goldie, Allen J Taylor, Peter Nguyen, Cody McCoy, Xue-Qiao Zhao, David Preiss

**Affiliations:** 1 BHF Glasgow Cardiovascular Research Centre, University of Glasgow, Glasgow, UK; 2 Medstar Research Institute, Washington Hospital Center, Washington DC, USA; 3 Division of Cardiology, Medstar Georgetown University Hospital, Washington DC, USA; 4 University of Tennessee Health and Science Center, College of Medicine, Memphis, Tennessee, USA; 5 Cardiovascular Atherosclerosis Research Laboratory, Division of Cardiology, University of Washington, Seattle, Washington, USA

## Abstract

**Objective:**

Previous studies have suggested that niacin treatment raises glucose levels in patients with diabetes and may increase the risk of developing diabetes. We undertook a meta-analysis of published and unpublished data from randomised trials to confirm whether an association exists between niacin and new-onset diabetes.

**Methods:**

We searched Medline, EMBASE and the Cochrane Central Register of Controlled Trials, from 1975 to 2014, for randomised controlled trials of niacin primarily designed to assess its effects on cardiovascular endpoints and cardiovascular surrogate markers. We included trials with ≥50 non-diabetic participants and average follow-up of ≥24 weeks. Published data were tabulated and unpublished data sought from investigators. We calculated risk ratios (RR) for new-onset diabetes with random-effects meta-analysis. Heterogeneity between trials was assessed using the I^2^ statistic.

**Results:**

In 11 trials with 26 340 non-diabetic participants, 1371 (725/13 121 assigned niacin; 646/13 219 assigned control) were diagnosed with diabetes during a weighted mean follow-up of 3.6 years. Niacin therapy was associated with a RR of 1.34 (95% CIs 1.21 to 1.49) for new-onset diabetes, with limited heterogeneity between trials (I^2^=0.0%, p=0.87). This equates to one additional case of diabetes per 43 (95% CI 30 to 70) initially non-diabetic individuals who are treated with niacin for 5 years. Results were consistent regardless of whether participants received background statin therapy (p for interaction=0.88) or combined therapy with laropiprant (p for interaction=0.52).

**Conclusions:**

Niacin therapy is associated with a moderately increased risk of developing diabetes regardless of background statin or combination laropiprant therapy.

## Introduction

Despite apparently beneficial effects on total-cholesterol and high-density lipoprotein (HDL) cholesterol, recently published data from major trials of the lipid-modifying agent niacin have shown no cardiovascular benefit when niacin is added to background statin therapy.^1^ ^2^ These results from the Atherothrombosis Intervention in Metabolic Syndrome with Low HDL/High Triglycerides: Impact on Global Health Outcomes (AIM-HIGH) trial and the Heart Protection Study 2-Treatment of HDL to Reduce the Incidence of Vascular Events (HPS2-THRIVE) trial contrast with findings from the Coronary Drug Project, conducted in the prestatin era, which showed a 17% reduction in non-fatal myocardial infarction (MI) or death from coronary causes.^3^ Major niacin clinical trials have provided insights into its effect on cardiovascular outcomes and its non-cardiovascular effects. While it has long been known that niacin therapy can cause cutaneous flushing, leading to various approaches to counteract this side effect, other detrimental effects have been noted.^2^ ^4^

One potentially important side effect known to occur on niacin is a rise in glucose levels in those with diabetes.^5^ Findings from a post-hoc analysis of the Coronary Drug Project suggested that this effect also occurred in those without diabetes, leading to an increase in the risk of developing diabetes.^6^ However, data regarding new-onset diabetes in trials of niacin therapy have not previously been systematically collected and pooled, and the recent publication of two major trials of niacin therapy presents an opportunity to do so.^1^ ^2^ It is also unclear whether any effect on new-onset diabetes is influenced by background statin therapy or whether treatment using the combination of niacin and laropiprant may have different effects compared with niacin therapy without laropiprant. Conclusive evidence that niacin adversely influences diabetes risk would be an important consideration when assessing risks and benefits of this treatment. We therefore conducted a meta-analysis of published and unpublished data to investigate niacin's effect on new-onset diabetes.

## Methods

### Search strategy and selection criteria

We gathered data from randomised controlled trials of niacin primarily designed to assess its impact on cardiovascular outcomes including both cardiovascular events and cardiovascular surrogate markers. We excluded trials that randomised fewer than 50 non-diabetic participants, those with a follow-up period of less than 24 weeks, and those conducted solely in patients with diabetes. Only trials comparing niacin to a relevant comparator were included: placebo, no therapy, standard care or a prespecified list of lipid-modifying agents, namely bile acid sequestrants or ezetimibe based on the rationale that these are considered unlikely to alter diabetes risk. We excluded any trials that directly compared niacin to statin therapy or fibrate therapy based on the rationale that these comparator agents may influence diabetes risk. Combination therapy trials were allowed as long as the other lipid-modifying therapy or therapies involved were balanced between the niacin arm and the control arm. Trials in which niacin was combined with laropiprant were included. Data for trials only comparing different doses of niacin without any placebo-treated or untreated arms were not considered relevant. Some trials included three or more arms and in such cases we only included those arms that met the criteria described above, and pooled them as required for analysis.

**Figure 1:**
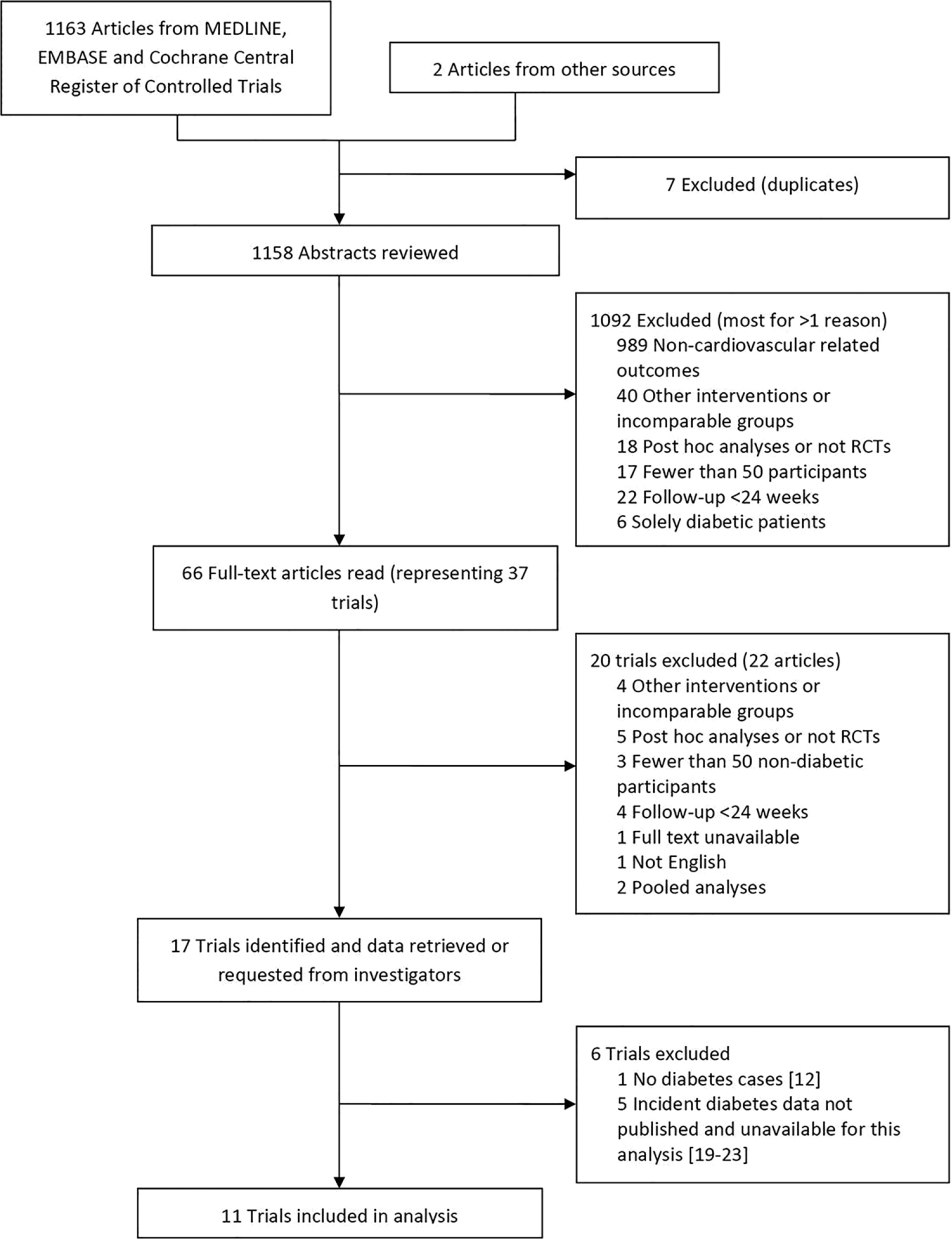
Flow diagram of literature search to identify randomised controlled niacin trials.

We searched Medline, EMBASE and the Cochrane Central Register of Controlled Trials for randomised controlled trials with the medical subject heading (MeSH) terms ‘niacin’, ‘nicotinic acid’ or ‘Niaspan’ as title words and keywords (see figure 1). We restricted our search to between the years 1975 and 2014, trials that involved adult patients, and reports that were published in English. We undertook our search on 18 June 2014, and identified 1163 reports. An additional two reports were added on 17 July 2014 as incident diabetes data became available for two major studies, namely AIM-HIGH and HPS2-THRIVE.^1^ ^2^ Reports were independently reviewed by two readers (CG and DP), and any discrepancies were resolved by consensus.

### Data sources

For the eight trials that had published data on new-onset diabetes,^2^ ^4^ ^6–11^ information on the number of non-diabetic participants at baseline and cases of incident diabetes were independently abstracted by two authors (CG and DP) and tabulated. One trial that had found no cases of new-onset diabetes was excluded.^12^ We contacted investigators from eight other trials meeting our search criteria in which incident diabetes data remained unpublished and, using a formal questionnaire sheet (see online supplementary eFigure 1), we received and included data from three of these trials.^13–15^ Eleven trials were therefore included in the final analysis, of which eight had previously published data for incident diabetes and three had not. For the three trials with unpublished data, incident diabetes data had not been analysed prior to our request. The study was conducted in accordance with PRISMA guidance.^16^

### Endpoints

A patient was considered to have developed diabetes during a trial if: there was an adverse event report of new-onset diabetes mellitus; the participant commenced oral diabetes medication or insulin; the participant had two post-baseline fasting plasma glucose values ≥7.0 mmol/L.

### Quality assessment

Two authors (CG and DP) independently evaluated the quality of each of the trials, using a well-established tool.^17^ Nine characteristics were assessed: randomisation, concealment of treatment allocation, baseline similarity of randomised groups, eligibility criteria, outcome assessor blinding, care provider blinding, patient blinding, availability of point estimates and measures of variability and intention to treat analysis. Assessment of these criteria allowed each trial to be awarded a Delphi score of 0–9 with a higher score indicating higher quality.

### Statistical analysis

To identify the potential effect of niacin on incident diabetes, we calculated an overall risk ratio (RR), with associated 95% CIs, as the ratio of cumulative incidence from the available data for all non-diabetic participants at baseline and for those who developed diabetes during trial follow-up. Study-specific RRs were pooled in a random-effect model meta-analysis. We assessed statistical heterogeneity across all studies using the I^2^ statistic, which is derived from Cochrane's Q ((Q−df/Q)×100) and provides a measure of the proportion of overall variation attributable to the heterogeneity between studies.

Despite using both published and unpublished data, we nonetheless formally investigated publication bias by producing a funnel plot and undertaking an Egger test. We additionally undertook sensitivity analyses, using the fixed-effect inverse-variance method, to compare trials that had and had not used laropiprant in combination with niacin, and to compare trials that had and had not used background statin treatment. We repeated the main analysis after excluding the largest trial and also performed an analysis using a fixed-effects model. All p values were two-sided, and p<0.05 was considered statistically significant. We analysed data with StataSE V.13 (StataCorp).

## Results

**Figure 2:**
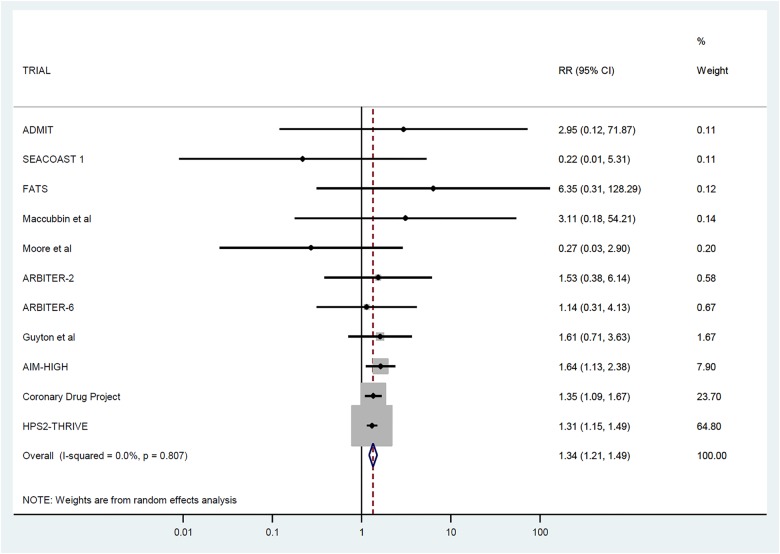
Association between niacin therapy and incident diabetes in 11 trials. (Ordered by weight contributed to meta-analysis.) AIM-HIGH, Atherothrombosis Intervention in Metabolic Syndrome with Low HDL/High Triglycerides: Impact on Global Health Outcomes; HPS2-THRIVE, Heart Protection Study 2-Treatment of HDL to Reduce the Incidence of Vascular Events.

The 11 trials included in the meta-analysis provided data on 26 340 participants without diabetes at baseline (baseline characteristics in table 1). Over a weighted mean follow-up duration of 3.6 years, 1375 (5.22%) were diagnosed with diabetes. Of those treated with niacin, 725 (5.53%) out of 13 121 participants developed diabetes while, on control treatment, 646 (4.89%) out of 13 219 developed diabetes. This represents a RR of 1.34 (95% CI 1.21 to 1.49) (see figure 2). Expressed in absolute terms, treating 43 (95% CI 30 to 70) initially non-diabetic individuals with niacin for 5 years would result in one additional case of diabetes being diagnosed compared with no treatment.

**Table 1:** Data for non-diabetic participants in 11 trials included in meta-analysis

Trial	Active vs control daily therapies	Non-DM patients/total cohort (%)	N (without DM) on niacin/control	Description	Follow-up (months)	Method of DM diagnosis	Mean BMI (kg/m^2^)	Mean age (years)	Frequency of within-trial FPG measurement
Coronary drug project^6^	Niacin 3 g vs placebo	3436/3906 (88)	988/2448	Men with previous MI	60	I, II, III	26.1	52.5	Yearly
FATS^7^	Niacin 4 g/colestipol 30 mg vs placebo or placebo/colestipol 30 mg	82/82 (100)	36/46	Men with high ApoB and family history of CHD	32	II	26.7	46.7	6 monthly
ADMIT^8^	Niacin 3 g vs placebo	343/468 (73)	173/170	Peripheral artery disease, with and without DM	11	II	27	65	6 weekly
ARBITER-2^13^	ERN 1 g vs placebo (background statin)	121/167 (72)	63/58	CHD and HDL <1.16 mmol/L	12	II, III	29.1*	67*	At 12 months
Moore *et al*^15^	ERN 2 g/atorvastatin±colsevelam 3.8 g vs placebo/atorvastatin	108/123 (88)	70/38	CHD or carotid atherosclerosis with high ApoB and LDL-c	12	I, II, III	29.6*	55*	At months 5 and 12
SEACOAST I^9^	ERN 1 g/simvastatin 20 mg or 2 g/20 mg vs simvastatin 20 mg	248/314 (79)	150/98	High CVD risk with mixed dyslipidaemia	4	Not stated	28.2*	56.6*	At week 24
Guyton *et al*^10^	ERN 2 g/ezetimibe 10 mg/simvastatin 20 mg vs ezetimibe 10 mg/ simvastatin 20 mg	798/942 (85)	569/229	Type IIa or Type IIIb hyperlipidaemia	16	I, II, III	30.1*	57.2*	4 weekly
Maccubbin *et al*^11^	ERN 2 g/LRPT 40 mg or ERN 2 g vs placebo (with or without background statin)	1356/1613 (84)	1124/232	Primary hypercholesterolaemia or mixed dyslipidaemia	4	I, II	29*	–	At week 24
ARBITER-6^14^	ERN 2 g vs ezetimibe 10 mg (background statin)	218/363 (60)	114 /104	CHD or high CHD-risk, with LDL <2.6 mmol/L and low HDL	14	II, III	30.9*	64.5*	At months 2, 8 and 14
AIM-HIGH^1^	ERN 1.5–2 g/simvastatin 40–80 mg vs placebo/simvastatin 40–80 mg (with or without ezetimibe 10 mg)	2256/3414 (66)	1130/1126	Atherosclerotic CVD and LDL <1.81 mmol/L	36	Not stated	–	63.7*	6 monthly
HPS2-THRIVE^2^	ERN 2 g/LRPT 40 mg/simvastatin 40 mg vs placebo/simvastatin 40 mg (with or without ezetimibe 10 mg)	17 374/25 673 (68)	8704/8670	Patients with CVD	47†	I, II	27.7*	64.9*	None
Total		26 340/37 065 (71)	13 121/13 219						

I=adverse event report or physician report; II=glucose lowering therapy; III=raised fasting plasma glucose (≥7.0 mmol/L) on two occasions.

*Data from total cohort (including diabetics at baseline).

‡Weighted mean follow-up.

AIM-HIGH, Atherothrombosis Intervention in Metabolic Syndrome with Low HDL/High Triglycerides: Impact on Global Health Outcomes; BMI, body mass index; CHD, coronary heart disease; CVD, cardiovascular disease; DM, diabetes mellitus; ERN, extended release niacin; FPG, fasting plasma glucose; HPS2-THRIVE, Heart Protection Study 2-Treatment of HDL to Reduce the Incidence of Vascular Events; HDL,high-density lipoprotein; LDL, low-density lipoprotein; LRPT, laropiprant; MI, myocardial infarction.

Heterogeneity across trial results was limited (0.0%, p=0.87). There was no evidence of publication bias according to either the Egger test (p=0.15) or funnel plot (see online supplementary eFigure 2). The trials were generally found to be of high quality (median Delphi score 9, range 6–9).

Sensitivity analyses demonstrated no difference between the trials that had combined laropiprant with niacin and the trials that had not (p=0.52 for interaction; see online supplementary eFigure 3). Similarly, no difference was observed when comparing the trials that had or had not used background statin treatment (p=0.84 for interaction; see online supplementary eFigure 4). We performed an analysis with the largest trial, HPS2-THRIVE (which provided 65% of the overall weighting of the main analysis), excluded. In the remaining 10 trials, the RR of new-onset diabetes on niacin was similarly elevated at 1.38 (95% CI 1.16 to 1.65). Finally, we repeated the meta-analysis using fixed-effects modelling, and the results were very similar to the main analysis, namely RR 1.35 (95% CI 1.21 to 1.50).

## Discussion

This comprehensive meta-analysis of published and unpublished data from randomised controlled trials of niacin demonstrates that niacin therapy led to a 34% increase in the risk of developing diabetes compared with placebo or standard care. These findings complement previous study reports that have shown deteriorations in glycaemic control in patients with diabetes on niacin. Results were also consistent regardless of the presence or absence of background statin therapy or concomitant laropiprant therapy.

The mechanism that explains niacin's detrimental effect on glycaemic control and diabetes risk remains unclear. The observation that statins also increase diabetes risk and that this appears to be an on-target effect^18^ suggests that a specific lipid-modifying effect warrants particular attention. The Mendelian randomisation approach taken by Swerdlow *et al* to establish whether the modest increase in diabetes risk on statins is an on-target or off-target effect may be applicable for niacin. The data from HPS2-THRIVE also confirm that clinically significant deteriorations in glucose control in patients with diabetes are substantially increased on niacin. In those known to have diabetes at baseline in this major trial, there was a 55% increase in serious disturbances in glucose control for patients with diabetes, most of whom required hospital admission as a result.^2^ These findings contrast with earlier and smaller trials with shorter follow-up which suggested that the detrimental effect of niacin on glucose may only be temporary and therefore of potentially limited clinical importance.^8^ ^10^ Additionally, the Coronary Drug Project showed that the relative risk of developing diabetes on niacin was similar regardless of whether patients had impaired fasting glycaemia (IFG) or normoglycaemia though the absolute risk was higher in IFG due to the increased rate of progression to diabetes.^6^

Even with the discovery that statins modestly increase diabetes risk, their cardiovascular benefits still greatly outweigh any such metabolic risk. With niacin, the risk:benefit ratio appears far less favourable. Recent major niacin trials have now confirmed a lack of cardiovascular benefit when niacin is added to statin therapy and increased risk of a variety of adverse events along with the expected increase in cutaneous flushing. In HPS2-THRIVE, gastrointestinal events, musculoskeletal events and infectious adverse events were increased on niacin.^2^ The AIM-HIGH trial recently released supplementary data detailing all adverse events seen during the trial. Here again, increases were seen in gastrointestinal events and infectious events compared with placebo.^4^ The results from HPS2-THRIVE and AIM-HIGH differ from the historical Coronary Drug Project, a clinical trial of niacin monotherapy which suggested modest cardiovascular benefit with similar effects across all glycaemic categories.^3^ ^19^ As a result of these disappointing cardiovascular trials, combination therapy of niacin with laropiprant has been withdrawn from the market. However, extended release niacin is still available for prescription in the USA in brand and generic versions.

One of the strengths of this analysis is that we were able to include data (published and unpublished) from almost all relevant niacin trials, thereby maximising our ability to confirm any effect. Five trials were not included in this meta-analysis, due to the unavailability of unpublished data^12^ ^20–24^ but these were relatively small studies whose inclusion would not have changed the main findings. These trials would have provided information on 634 non-diabetic trial participants, only 2.4% of the total cohort. Additionally, there was a very low level of heterogeneity across trial results. Although no large-scale trials of niacin are likely to be undertaken in the future, it would appear sensible to include new-onset diabetes as a prespecified endpoint in major future trials of lipid-modifying agents.

An important weakness of our analysis is that no trials were specifically designed to assess new-onset diabetes, and new-onset diabetes was only a prespecified endpoint in one major trial. We were unable to compare the effects of niacin on diabetes risk and cardiovascular events respectively to provide a fuller clinical context, and we could also not compare risks across subgroups of diabetes risk factors like fasting glucose and body mass index (BMI). Analyses were conducted without access to individual participant data. It is also unclear if niacin therapy alters microvascular disease risk over time in those who develop diabetes. In order to estimate the average follow-up, we assumed the median approximated to the arithmetic mean for some trials and, in several trials, the quoted mean BMI and mean age at baseline are averages taken from the full cohort because specific data for non-diabetic participants were unavailable.

In summary, this analysis confirms that niacin increases the risk of developing diabetes by about 35% and that this risk occurs regardless of whether concomitant statin therapy or combination therapy with laropiprant is used.

**Key messages**

**What is already known on this subject?**

- Previous studies have suggested that niacin therapy may have a detrimental effect on glycaemic control in those with diabetes, and may increase the risk of developing diabetes. Data from all niacin trials have not previously been pooled to systematically examine the risk of niacin's risk on new-onset diabetes, and it is also unknown whether any relationship may be affected by concomitant statin (statins have been shown to have a diabetogenic effect themselves) or laropiprant (DP1 antagonist) therapy.

**What might this study add?**

- We systematically collected and pooled data regarding new-onset diabetes from 11 trials of niacin, involving 26 340 patients. Niacin therapy led to a 34% higher risk of developing diabetes. This effect was not influenced by the presence or absence of statin therapy or by coadministration with laropiprant.

**How might this impact on clinical practice?**

- Failure in two major clinical trials (AIM HIGH, HPS2-THRIVE) when niacin was added to statin therapy led to the withdrawal of the drug in Europe. However, the other brand and generic versions are still in use in the USA and elsewhere, and proponents of niacin argue that the cardiovascular benefit of niacin monotherapy observed in the Coronary Drug Project suggests that niacin should still be used in some patients. Our data suggest that new-onset diabetes is a major concern with this agent.

## Supplementary Material

Web supplement
