# Supplementary material for: Niacin therapy and the risk of new-onset diabetes: a meta-analysis of randomised controlled trials
Source: Heart. 2015 Sep 14;102(3):198–203. doi: 10.1136/heartjnl-2015-308055 (PMC4752613; doi:10.1136/heartjnl-2015-308055)

Supplementary material

Goldie C et al, Niacin therapy and the risk of new-onset diabetes: a meta-analysis of randomised controlled trials

eFigure 1. Data request sheet for niacin trials (page 2)

eFigure 2. Funnel plot of the eleven trials with 95% confidence intervals (page 3)

eFigure 3. Sensitivity analysis on trials that did/ did not use laropiprant (page 4)

eFigure 4. Sensitivity analysis on trials that did/ did not use background statin therapy (page 5)

eFigure 1. Data request sheet for niacin trials

From David Preiss
University of Glasgow

Data request for niacin incident diabetes meta-analysis:

Thank you for approving our request to join this meta-analysis. The following is a summary of the data required to enable us to incorporate data from your trial into the meta-analysis. We do not require all data; the most important is for the numbers developing diabetes. Some studies have access to all three methods to diagnose diabetes and some to only one of the three; this is not problematic and will be highlighted in the resulting manuscript.

Essential data

1. Total number of non-DM subjects at baseline:
    a. Niacin:
    b. Placebo:

2. Methods of diagnosis of diabetes – which of the following were available? (please state *yes / no* as appropriate)
    a. Physician reported (i.e adverse event report): ___
    b. Commencement of oral diabetes medication or insulin: ___
    c. Two elevated fasting plasma glucose values (≥126mg/dL or 7.0mmol/L): ___

3. Number developing diabetes in each group:
    a. Niacin: ___
    b. Placebo: ___

Additional data

4. Age (mean) of all non-DM participants at baseline: ___

5. BMI (mean) of all non-DM participants at baseline: ___

6. Follow up duration (mean): ___

eFigure 2. Funnel plot of the eleven trials with 95% confidence intervals

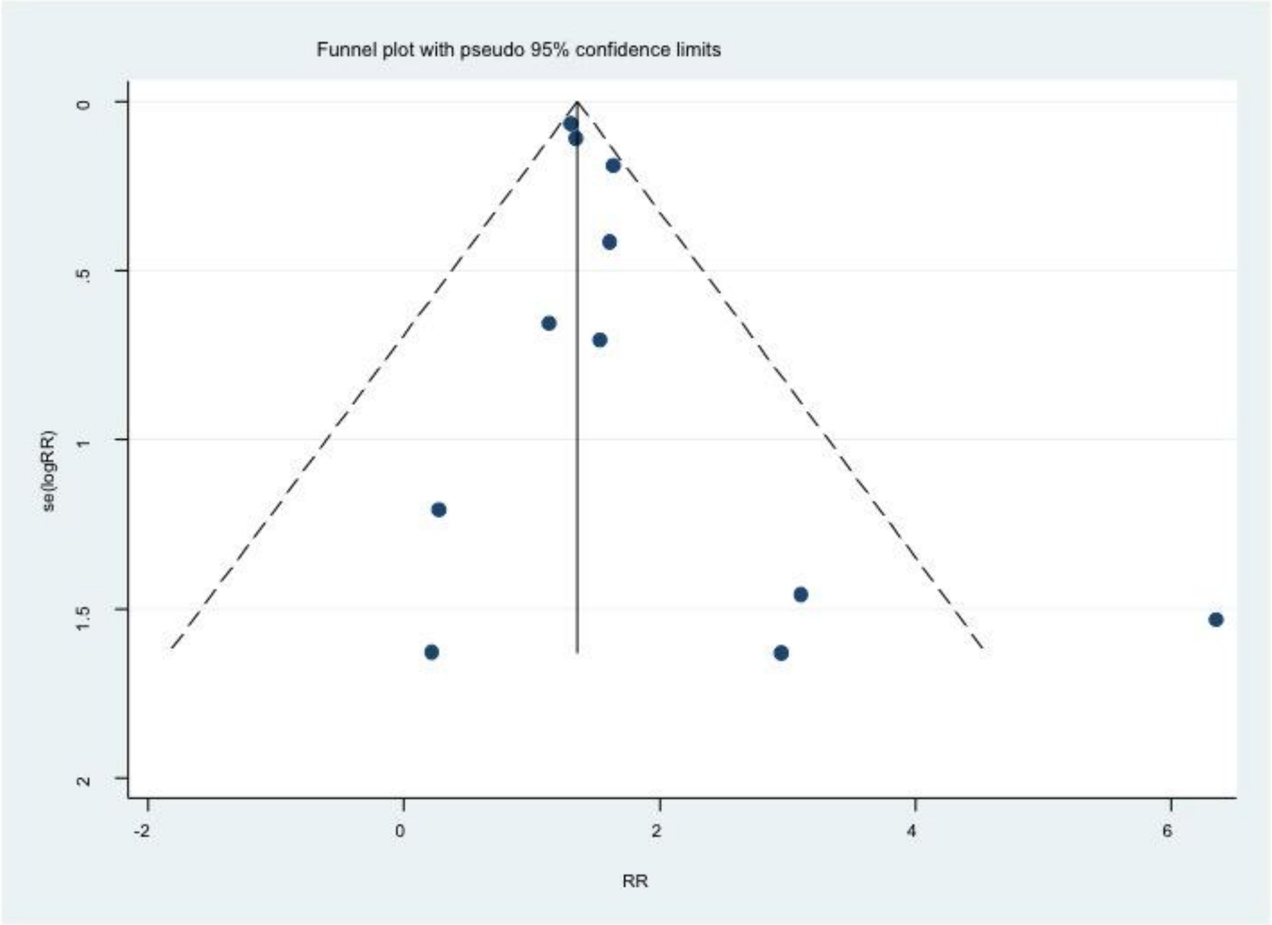

eFigure 3. Sensitivity analysis on trials that did/ did not use laropiprant

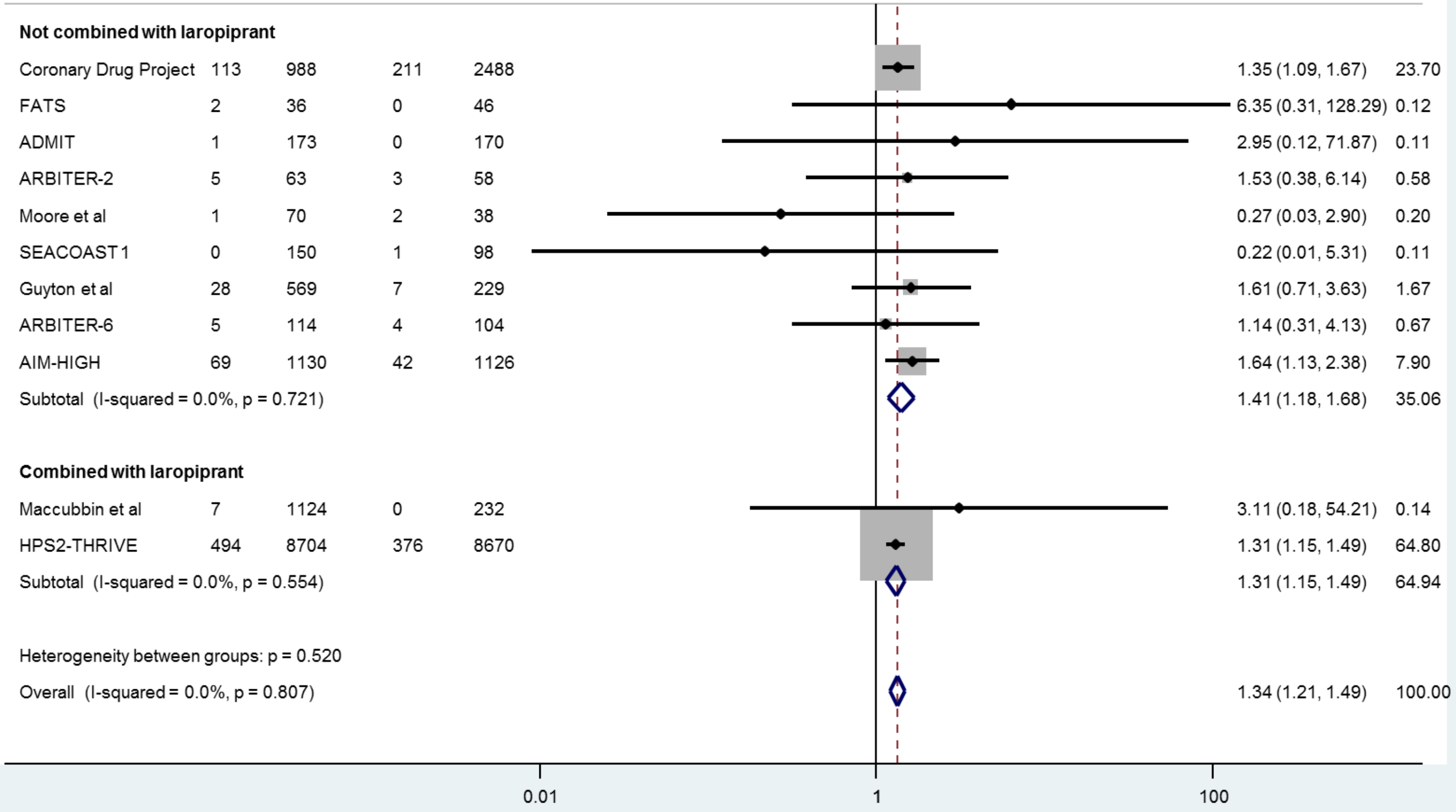

TRIAL	DM on niacin	Total on niacin	DM on control	Total on control	RR (95% CI)	% Weight
**Not combined with laropiprant**						
Coronary Drug Project	113	988	211	2488	1.35 (1.09, 1.67)	23.70
FATS	2	36	0	46	6.35 (0.31, 128.29)	0.12
ADMIT	1	173	0	170	2.95 (0.12, 71.87)	0.11
ARBITER-2	5	63	3	58	1.53 (0.38, 6.14)	0.58
Moore et al	1	70	2	38	0.27 (0.03, 2.90)	0.20
SEACOAST 1	0	150	1	98	0.22 (0.01, 5.31)	0.11
Guyton et al	28	569	7	229	1.61 (0.71, 3.63)	1.67
ARBITER-6	5	114	4	104	1.14 (0.31, 4.13)	0.67
AIM-HIGH	69	1130	42	1126	1.64 (1.13, 2.38)	7.90
Subtotal (I-squared = 0.0%, p = 0.721)					1.41 (1.18, 1.68)	35.06
**Combined with laropiprant**						
Maccubbin et al	7	1124	0	232	3.11 (0.18, 54.21)	0.14
HPS2-THRIVE	494	8704	376	8670	1.31 (1.15, 1.49)	64.80
Subtotal (I-squared = 0.0%, p = 0.554)					1.31 (1.15, 1.49)	64.94
Heterogeneity between groups: p = 0.520						
Overall (I-squared = 0.0%, p = 0.807)					1.34 (1.21, 1.49)	100.00

eFigure 4. Sensitivity analysis on trials that did/ did not use background statin therapy

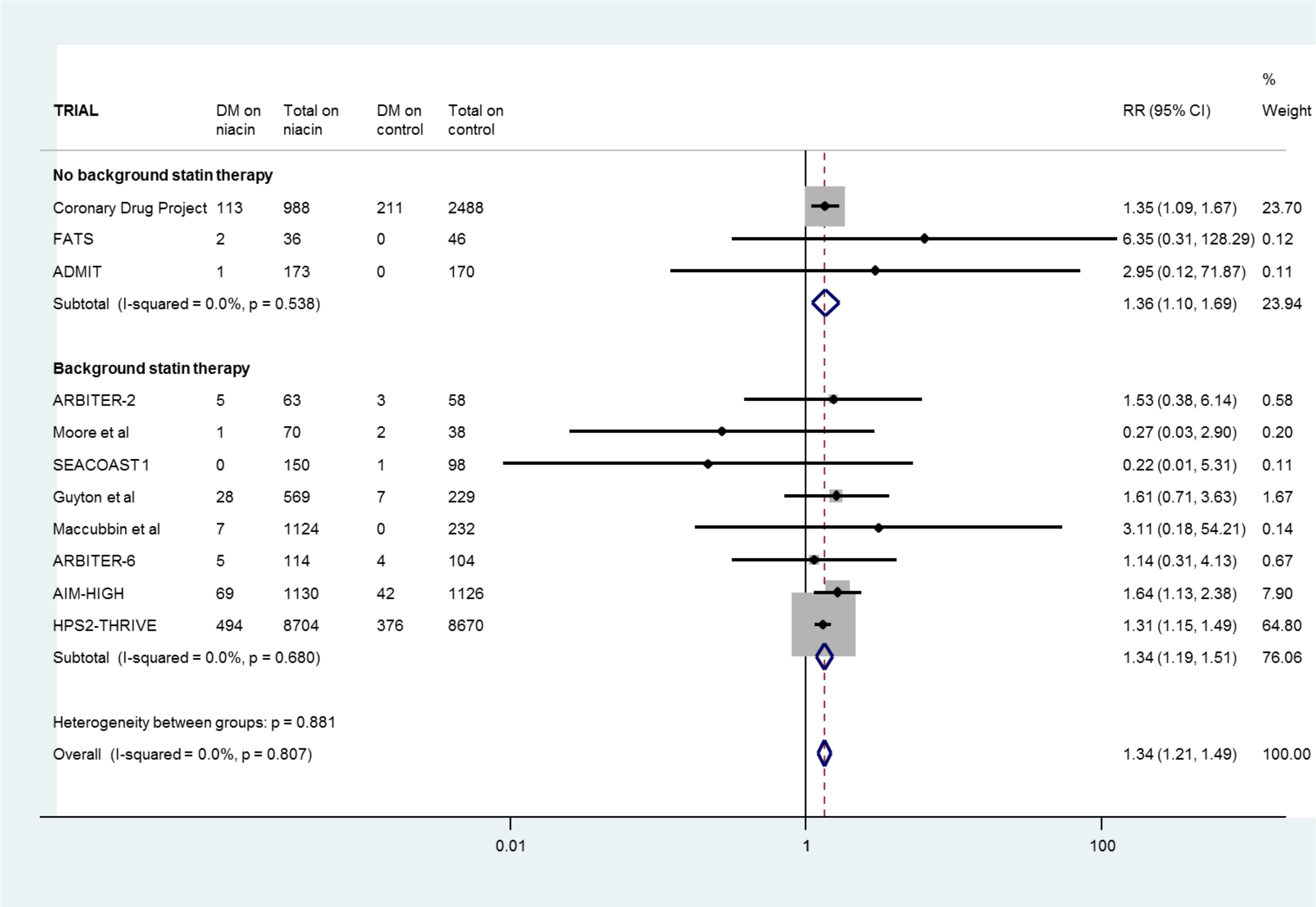

Supplement: Web supplement [file heartjnl-2015-308055-s1.pdf]
